# Development and Validation of a Smartphone Application for Telenutrition in Patients with Inflammatory Bowel Disease

**DOI:** 10.3390/diagnostics12102482

**Published:** 2022-10-13

**Authors:** Arti Gupta, Namrata Singh, Divya Madan, Mariyam Farooqui, Neha Singh, David Mathew Thomas, Bhaskar Kante, Mukesh Singh, Shubi Virmani, Mehak Verma, Aditya Bajaj, Manasvini Markandey, Peeyush Kumar, Sudheer Kumar Vuyyuru, Pabitra Sahu, Nitika Monga, Govind Makharia, Saurabh Kedia, Vineet Ahuja

**Affiliations:** 1 Department of Gastroenterology and Human Nutrition Unit, All India Institute of Medical Sciences, New Delhi 110029, India; 2 Indian Council of Medical Research, New Delhi 110029, India

**Keywords:** diet research tool, IBD mobile app, diet counselling, nutritional assessment

## Abstract

The use of smartphone-based applications as a telenutrition tool could redefine the nutritional management of IBD. We developed and validated a digital health platform in the form of a smartphone application for the nutritional assessment of IBD patients. Our team of gastroenterologists and dieticians at the All-India Institute of Medical Sciences, New Delhi developed a smartphone application titled IBD NutriCare, which was made available in both Android and iOS interfaces in English and seven other Indian languages. The application includes >650 Indian recipes and provides subjective global assessment and IBD clinical activity scores in a patient-friendly manner. The utility of the smartphone app was validated in comparison with the traditional 24-h dietary recall method. A total of 49 IBD patients were enrolled in the study. The mean difference in energy intake between the two dietary assessment methods was −4.776 kJ (95% LOA, range −417.916–408.365 kJ). A total of 94% of patients found the smartphone application convenient and acceptable in comparison to the recall method for dietary assessment. Bland–Altman plots showed a good level of agreement for nutrients and food groups between the two methods. Telenutrition in the form of a smartphone application helps in real-time tracking of dietary details of IBD patients, thus making appropriate interventions and large-scale data acquisition feasible.

## 1. Introduction

The burden of inflammatory bowel disease (IBD) in India has had an upward trajectory in recent years with disease rates paralleling the West [1,2]. Plausible explanations could be changes in dietary practices and environmental perturbations which come under the umbrella of the “Hygiene Hypothesis” [3,4,5]. Interestingly, the microbiome signature of Indian IBD patients mirror that of the diseased population in the West. Dietary influence on the pathogenesis of IBD cannot be overlooked in the Indian scenario where the at-risk genetic loci and family history play a lesser contributory role [6]. A sea change has occurred wherein dietary patterns from the past, which stressed high fiber intake and natural food sources for nutrient requirements, have given way to modernized food processing, and usage of food additives and emulsifiers, which have all harmed gut homeostasis leading to dysbiosis and a pro-inflammatory state [7,8]. Dietary interventions thus play a key role in modifying disease course in IBD patients [9,10,11,12].

There exists a vast gap between the theoretically proposed dietary specifications in IBD and their clinical implementation in day-to-day practice. Individualized dietary formulation, abolishing misconceptions in dietary beliefs and practices, and ensuring adherence to an anti-inflammatory diet could be quintessential in the achievement of deep remission in IBD [13]. Thus, the role of a dietician or a nutritionist cannot be undermined in attaining these targets. Traditional dietary assessment tools are retrospective and include pitfalls such as recall bias and are mostly time-consuming [14]. The Indian IBD scenario poses additional challenges in terms of heterogeneity of the population affected by the disease. Demographic, socioeconomic, cultural, and language barriers hinder in formalizing a unified dietary approach. With a predicted one billion smartphone users by 2026, digital platforms are key in redefining dietary interventions [15]. Smartphone-based applications coupled with the advent of artificial intelligence have made vast in-roads in revolutionizing nutrition and dietary goals for the common man, who may not have the luxury of availing a personalized dietician or access to routine clinical appointments. Telenutrition is an evolving field that provides real-time dietary assessment. Early identification of dietary triggers helps in formulating personalized dietary interventions for patient which may positively impact their disease course towards attaining clinical remission. Overall, it automates the process of large-scale data collection which otherwise would be labor- and time-intensive [16,17,18]. Although a plethora of diet and nutrition apps have flooded the Play Store and iOS platforms, there are yet no dedicated digital interfaces catering to the needs of IBD patients. Considering the enormous disease burden and the gaps in providing dietary interventions, we designed a smartphone-based application—IBD NutriCare—for the Indian IBD population and subsequently validated this against the traditional 24-h dietary recall method of nutritional assessment. This app aims to provide a personalized patient profile with database containing demographics, medications, daily dietary intake, clinical symptoms, and disease scores. The goals were primarily focused on formulating a personalized telenutrition counseling and ease of large-scale data acquisition, which could strengthen the clinical practice of dietary management in IBD patients in a resource-limited setting like India.

## 2. Materials and Methods

### 2.1. Development of the Smartphone Application (App)

The entire process of app development ranged over 19 months, from September 2019 to March 2021. A literature search was carried out regarding the availability of food tracking apps in the Android Play Store for IBD patients. The Android Playstore indeed had apps available for IBD patients albeit based on Western diet patterns, and none had Indian recipes catering to the requirement of the Indian IBD population.

The app development process took place in two phases:

Defining the scope of the app: In the first phase, extensive patient interviews were conducted by four dietitians in the IBD clinic. Brainstorming sessions within the core team, comprising of gastroenterologists and dietitians, were carried out to decide the languages, scope (i.e., amount of input/output information/advice from the app), serving sizes, disease related features such as frequently asked questions (FAQ), nutritional assessment, clinical disease activity scores, and formats of the user interface of the app to achieve the desired objectives of the dietary assessment and longitudinal data extraction for research.

Technical development: A team of application developers, gastroenterologists, and dieticians worked in close collaboration during the second phase of the design and development of the IBD NutriCare app. A preliminary prototype was designed with a graphical user interface appealing to the Indian population. More than 650 Indian food recipes with their nutrient values (as given in the Indian Food Composition Table (IFCT), 2017) were included in the database with permission from the National Institute of Nutrition (NIN), Hyderabad. The app was developed for availability in Android as well as iOS platforms using Java and Swift programming languages, respectively. The programming interface was supported by Google, and the database was developed using MS-SQL (Microsoft Structured Query Language). Nutrient information and questionnaires were integrated into the web pages.

**Figure 1:**
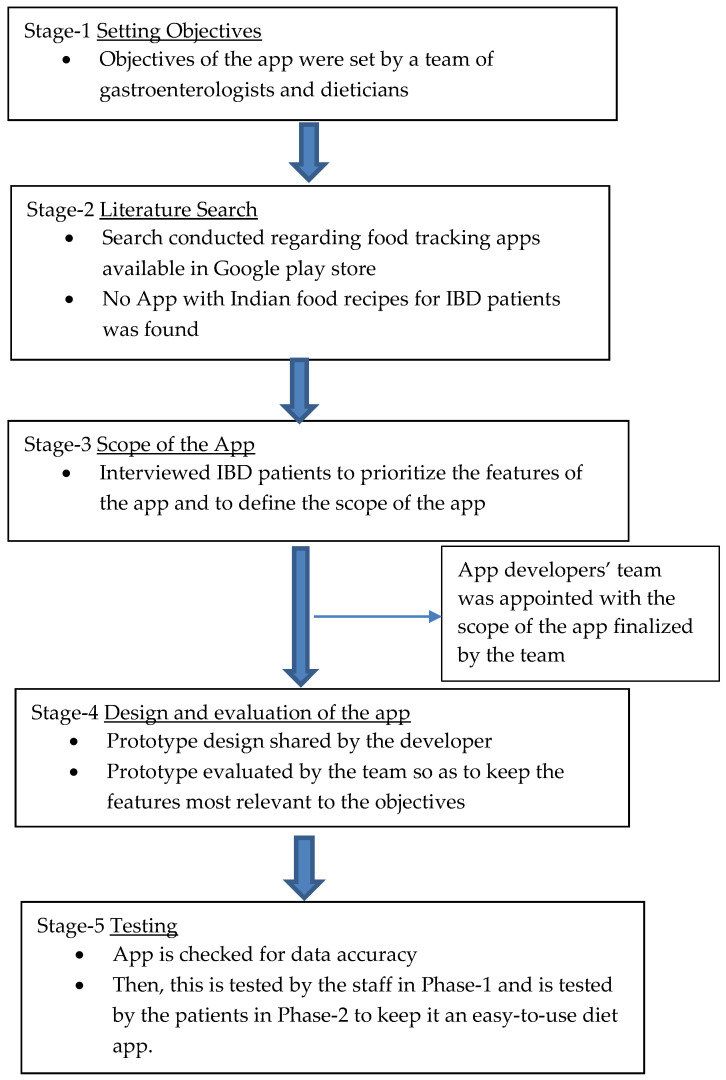
Flow chart of the IBD NutriCare App development.

A web-based application was created through a secure link between the mobile app and the web app to facilitate data retrieval for research purposes. The web app would allow researchers to review and edit the diet data entered by the patients as needed. The team tested the app regularly, and meetings were conducted to evaluate the application’s efficacy, ease of use, and utility. A team of dietitians evaluated the formats and confirmed the accuracy of the database. Data security was ensured by limiting access to the web application to the IBD team members. A pilot test was conducted amongst staff and select patients to assess the heterogeneity of the recipe list and the usage logistics. Figure 1 outlines the flowchart of the app development.

### 2.2. App Validation

After the app development, a study was conducted to validate the app as a tool to collect diet data for research purposes. The nutritional values from the app were compared against those collected using the traditional 24-h diet-recall method. Ethics clearance was obtained from the Institutional Ethics Committee (IEC/226/4/2021).

Study design: Prospective observational study.

Patients: Patients with IBD who were monitored at the IBD clinic of All India Institute of Medial Sciences, Delhi, irrespective of their disease extent/activity, aged 18–75 years, and with access to a smartphone, were screened and recruited from March to June 2021. The patients who required hospitalization and had undergone IBD-related surgery, and/or those unable to use a mobile app were excluded.

App-based diet entry: The dieticians registered the patients in the study using the web app, and their demographic details were recorded. The smartphone application was available on both Android and iOS platforms. A tutorial video was provided for every application user with explanations to common queries. The app features, and serving sizes used were also explained to the patients through a video call/at-clinic visit. A sample diet entry was demonstrated to every user by the concerned dietician at the clinic. The dieticians supervised the data entries in the first week to help patients familiarise themselves with the app. Patients were asked to continue adding diet data in the subsequent weeks in the app portal. At the end of 4 weeks, a questionnaire regarding the acceptability of the smartphone application was handed out which they were required to complete. The web app was used to generate reports containing patients’ dietary intake for a period of three days during which corresponding 24-h dietary recall was also done.

24-h diet recall: Patients were telephonically contacted during the second week on randomly selected three days to obtain a 24-hr diet recall. The standard practice took about 20–25 min to complete one recall. Patients were requested to give an account of their diet as per meal timings. Food items that often tend to get overlooked such as savoury snacks, sweets, and fruits were given special mention during data collection as standard practice. The consumption of oil/ghee was separately recorded to estimate its intake. To ascertain objectivity in the description of portions, same bowls, glasses, and Indian bread (chapatti) were shown to the subjects as used in the app. The meal recipes were converted to raw food items by the dietitians and nutrient intake values were calculated using “DietCal” software (based on IFCT, 2017).

### 2.3. Data Pre-Processing

Data entries in the app were monitored for errors and incorrect data entries. Typing errors were corrected after clarifying with the subjects. The changes were kept to the lowest minimum to assess the true validity of the app against the 24-h recall method.

### 2.4. Data Analysis

The nutrient/food group intake during the three days for which 24-h recall was taken was used for quantitative data analysis. Mean ± SD of the nutrients (energy, protein, carbohydrate, total fats, total fiber) and food groups (cereals and millets, pulses and legumes, vegetables, fruits, and milk/milk products) were calculated for all the patients based on the data entered in the IBD NutriCare app and 24-h recall method. Inter-class correlation coefficients were computed to validate the IBD NutriCare app against the 24-h recalls. Bland–Altman plots were constructed to assess the agreement between the IBD NutriCare app and 24-h recalls for the mean nutrients and food groups. IBM SPSS Statistics version 24 and Stata version 12.1 was used for all statistical analyses, and a *p*-value < 0.05 was considered significant.

## 3. Results

### 3.1. IBD NutriCare Mobile App

#### 3.1.1. IBD NutriCare Mobile App—For the Patients

IBD NutriCare app is a free-to-download app from any Android or iOS store via an OTP-enabled process. The registration requires entry of demographic parameters as well as height and weight for calculating the recommended nutrient values. The app is available in English, Hindi, Marathi, Telugu, Gujarati, Tamil, Malayalam, and Bengali, languages covering major geographical regions across India.

The key features of the app are:

A comparison of recommended energy and protein intake as per height, weight, and age (Harris–Benedict formula) with actual intake displayed graphically. Calorie consumption as per meal pattern was made available in a pie-chart format.

Indian food recipes covering regional dishes with their colloquial names as per commonly used serving sizes were displayed. If a patient encountered difficulty in finding a recipe of their choice, a provision was made whereby they could notify regarding its unavailability in the app itself. General dietary recommendations for IBD as per disease condition e.g., flare, remission, strictures along with frequently asked questions were also incorporated. 

Indices of clinical disease activity such as Simple Clinical Colitis Activity Index score (SCCAI) for ulcerative colitis (UC) [19]/Harvey-Bradshaw Index score (HBI) for Crohn’s Disease (CD) [20], and Subjective Global Assessment (SGA) [21] category were graphically displayed to help create disease awareness amongst patients and monitor their clinical course.

The app did not provide any automated suggestions. However, dieticians could regularly monitor the dietary intake based on the data obtained from the application which was accessible through the webpage. 

**Figure 2:**
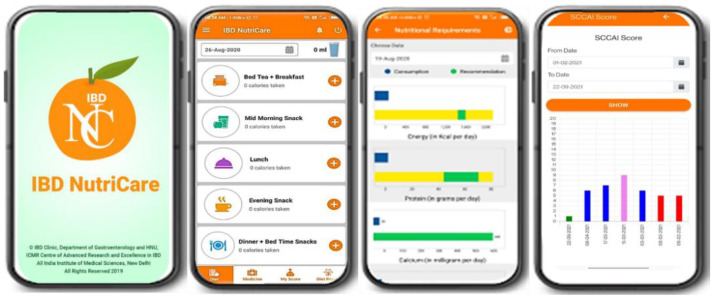
Screenshots of the features of the IBD NutriCare App.

The application also incorporated the medications which was specific to each patient in their profile along with their dietary parameters. Figure 2 displays a few screenshots of the IBD NutriCare app.

#### 3.1.2. IBD NutriCare Mobile App—For the Researchers

All the data parameters entered by the patients could be accessed by the re-searchers on the web app for data analysis. Any number of excel sheets containing food group or nutrient intake could be created simultaneously in the web app and the data could be compared with clinical parameters. Researchers could classify food items as per their research objectives and carry out analysis accordingly. A master report comprising all the variables and average diet data values for analysis could be exported in excel format. Personalized notifications could also be sent at any point of time to the patients through Webapp if erroneous or missing data entries were observed.

### 3.2. App Validation Study

A total of 70 IBD patients in our clinic consented to participate in the study, of which 49 completed at least 14 days of diet entry in 4 weeks in the diet app and a 24-h dietary recall. The remaining were excluded from analysis due to various reasons (16 patients had incomplete entries, 4 patients had incorrect entries, and one could not complete due to disease flare).

#### 3.2.1. Baseline Characteristics

The majority of patients had ulcerative colitis (77.5%), with Crohn’s disease noted in 12.2% and IBD-unclassified in 10.2%. Mean BMI was 20.6 ± 3.4 kg/m^2^, 30.6% of patients had a BMI exceeding the normal Asian standards (overweight or obese), while 32.6% were undernourished. The mean age of the cohort was 32.4 ± 1.2 years among which 75.5% were males. Average data-entry days recorded in the web application over 4 weeks were calculated to be 15.7 days. The baseline demographics of the patients included in the study are outlined in Table 1.

#### 3.2.2. Diet App Based vs. 24-hr Diet Recall Assessment

The IBD NutriCare app displayed a reasonably good accuracy as compared to the 24-h recall method as the mean difference (at 95% CI) in all food components was small and none were found to be statistically significant (*p* > 0.05) (Table 2). The difference in mean ± SD between the two methods was found to be higher in vegetables (6.88 ± 35.77), cereal and millets (5 ± 97.18), and fruits (−14.27 ± 67.27). The comparison of the 3-day average intake of nutrients and food group between the two methods is given in Table 2.

Intra-class correlation coefficients (ICC) for protein (0.93), fat (0.99), carbohydrates (0.93), energy (0.94), fiber (0.94), cereal and millets (0.91), fruits (0.93), milk/milk products (0.94), and grains and legumes (0.91) were excellent, while that for vegetables (0.79) was moderate, with statistically significant agreement between both methods. Overall, the *p*-value for all nutrients and food groups intake was <0.001, indicating significant agreement between the two methods (Table 2).

**Figure 3:**
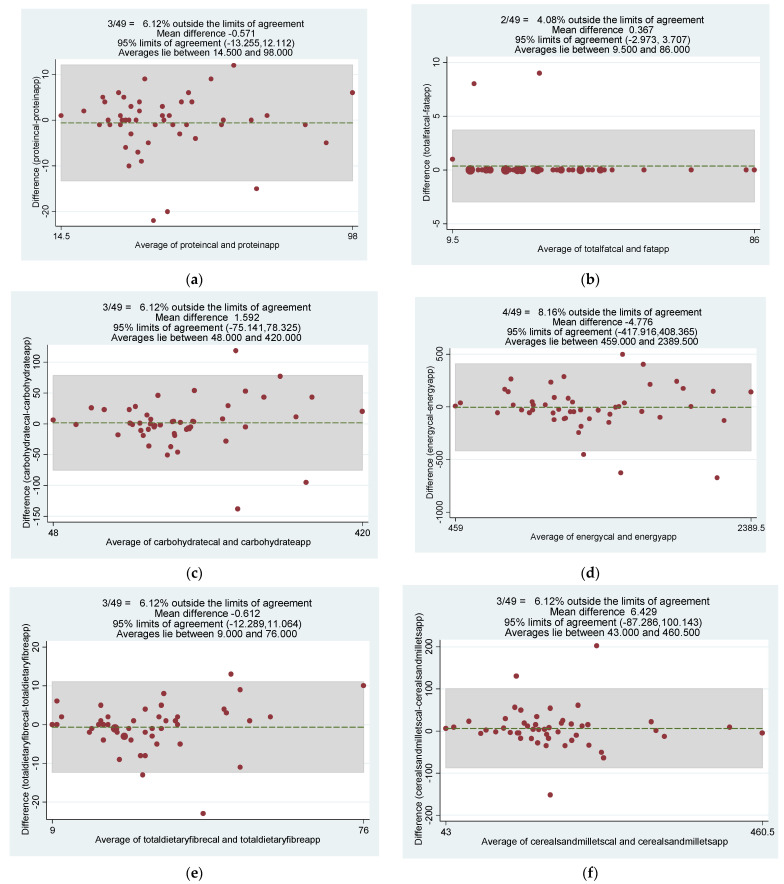

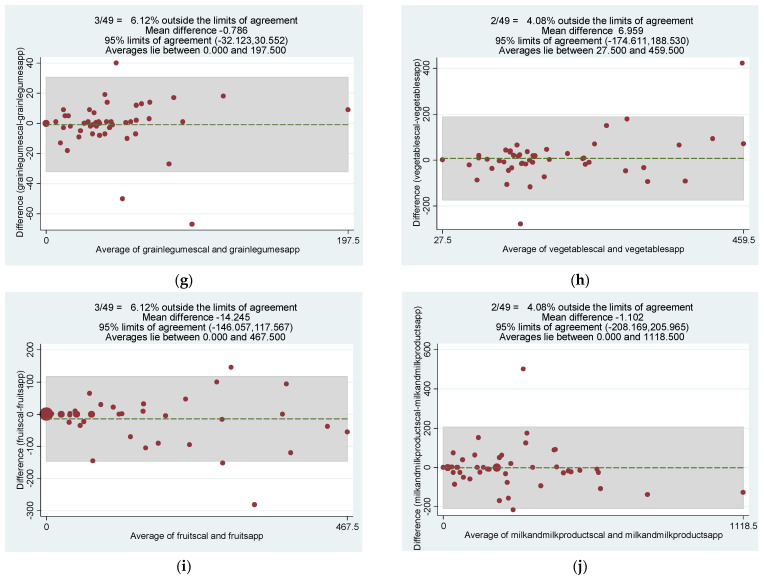
Bland–Altmann Plots showing the level of agreement between two methods of diet data collection (1) 24-h diet recall and (2) IBD NutriCare mobile app at 95% CI. (**a**) Protein; (**b**) fat; (**c**) carbohydrates; (**d**) energy; (**e**) total dietary fiber; (**f**) cereals and millets; (**g**) grains and legumes; (**h**) vegetables; (**i**) fruits; (**j**) milk/milk products.

Bland–Altman plots analysis was carried out for individual categories, comparing the two methods of assessment. The mean difference in energy intake between the two methods was −4.776 kJ with 95% LOA ranging from −417.916–408.365 kJ. Results showed good agreement between the app-based assessment compared with recall method (Figure 3).

#### 3.2.3. Acceptability of the App

Patients were asked to fill out a feedback questionnaire on the IBD NutriCare app to assess the acceptability of the interface. (Table 3). 94% of patients found the data entry in the app convenient and acceptable. 88% of respondents affirmed that this digital health platform was fairly accurate in estimating their food, and portion sizes and found it user-friendly, and saved a substantial portion of time spent in clinical visits trying manual recall of dietary items consumed. 16% of patients however had difficulty in identifying their food items or groups. When given a preference regarding their choice of diet assessment, 90% preferred using the app compared to manual recall, even though 90% did not have a prior experience with a similar interface

## 4. Discussion

This article outlines the systematic development of a first-of-its-kind smartphone application (IBD NutriCare) dedicated to the IBD population in India. Following the design and development of the app, we validated its feasibility and acceptability amongst our patients in the IBD clinic by comparing it with the standard 24-h dietary recall. Out of the 49 patients who consented to participate in the study, 90% (44/49) opined that the smartphone-based interface was more acceptable in terms of data entry, creating disease awareness, reducing time consumption for dietary recall, and maintaining adherence to dietary counsel and adjustments, in comparison to routine clinical interviews using the pen-paper mode. The correlations between IBD NutriCare and the 24-h recall for macronutrients and food groups intake were good to excellent (r 0.79 to 0.99) and the Bland–Altman plots showed a good level of agreement between the two methods. The availability of the app in English and seven other Indian languages further broadens its scope, and the ability to assess nutritional status, demographics, and disease dynamics makes it a novel yet unique platform for IBD care. Having a web-based application at the backend further enhances its ability as a research tool, as a large amount of data can be exported for advanced analysis and interpretation without undue burden on the researcher and the participant. A total of 88% of respondents to the feedback questionnaire (administered after 4 weeks of app use) reported wholesome communication with their gastroenterologists and dieticians, and better work productivity.

The utility of telemedicine in improving outcomes in IBD has been well-elaborated in Western literature. In a study which assessed the acceptance of a remote automated telemanagement system in UC patients by Raymond Cross et al, it was found that there was a high level of acceptance amongst subjects, with improved self-efficacy of patients, medication adherence, and clinical parameters [22]. In a review on digital health apps for clinical care in inflammatory bowel disease by Yin et al., a total of 11 apps were identified. The majority did have significant benefits in improving the quality of life, treatment adherence, and disease outcomes, and none reported any negative outcomes [23]. In another study by Ambrosini, G.L. et al, comparing smartphone applications with 24-h dietary recalls, 83% preferred a digital interface and the average difference in energy intake was 268 kJ/d [24]. My Meal Mate (MMM) was one of the pioneer smartphone apps designed for the UK population in assisting weight loss management. They validated the app measurements against the traditional 24-h dietary recalls in a sample size of 50 volunteers where Bland–Altman analysis was used to assess the agreement between the methods. The average difference in energy intake between methods was 218 kJ/d, with LOA ranging from −2434 kJ to 2022 kJ [14]. It is key that patients be made aware of the role played by nutrition in the pathogenesis of IBD. Thus, smartphone applications and telenutrition has been on the horizon for long, but its potential has never been objectively elucidated in a resource constrained setting like India. The consumption of processed foods and ready-to-eat consumables is becoming increasingly popular. “Fast food” apps, now with assistance from AI input, are now custom-made to allure and entice the common man’s appetite. All of these carry a three or four-fold risk of developing IBD [25]. Shoda R, et al. conducted an epidemiological analysis of Crohn’s disease in Japan, and the strongest independent factor which correlated with the incidence of Crohn’s was an increased dietary intake of animal fat [26].

The challenges in establishing a unified dietary protocol for IBD patients are many and varied. The first hurdle would be to maintain a proper dietary data record. It is virtually impossible to conduct a double-blind study with dietary items. Moreover, changing food habits in patients is more difficult to achieve than convincing the need for biologicals. A patient’s consistency to adhere to a prescribed diet is another indicator of clinical success. Numerous studies have highlighted patient preference for smartphone-based dietary assessment over traditional methods [14,27]. Conventional methods are often cumbersome, labor intensive, and subject to many inadequacies in the data retrieved. Smartphone apps provide real-time data which alert the nutritionist or dietician regarding adherence issues, erroneous entries, and incorrect information, thus helping initiate early and timely dietary interventions and formulating individualized diet plans. Clinical visits are often stressful and eliciting a proper dietary recall through interviews is challenging. The IBD NutriCare app and similar apps offer the advantage of more quality time spent with the participant in counseling and offering dietary adjustments which could have a great impact on disease outcomes [28,29].

As the adage goes, the strength of a chain is in its weakest link; this seems aptly suited for the Indian IBD scenario regarding the role played by a nutritionist in the IBD care pathway. The accessibility and availability of a dedicated nutritionist for IBD patients are lacking. Apart from individualized counseling, ensuring compliance to medications, assessing refeeding risk, and measurement of sarcopenic parameters can all be accomplished by a dedicated dietician, which would bridge the existing gulf between the bench and the bedside [30]. The IBD NutriCare diet app is constituted in a manner that includes the majority of the recipes prepared in Indian kitchens. With the largest diaspora population in the world, at 18 million people [31], this smartphone platform would transcend geographical, cultural, and ethnic backgrounds to track the Indian IBD population, concerning their diet-specific needs and interventions.

Patient education, availability of smartphones, and adherence to dietary advice are the key issues that limit its clinical use. Heterogeneity in patient populations that differ in socio-economic and educational backgrounds stands as a barrier to uniformity in app use and data retrieval.

To conclude, the digital health platform (IBD NutriCare) for telenutrition provides a potential tool for improving patient care in IBD. This study validated the app against the traditional recall method of diet assessment, with excellent acceptability rates. The database provided by the app could be utilized to bridge the existing gaps regarding our knowledge of dietary interventions in IBD. It makes feasible multicentric longitudinal dietary intervention studies, that could provide us with robust high-quality data which otherwise would be cumbersome with traditional dietary assessment methods.

## Figures and Tables

**Table 1:** Baseline demographics of patients enrolled in validation study (*n* = 49).

Parameter	Result *n* (%)
**Mean Age** at onset (years, SD)	32.4 ±11.2
**Gender**	
Male	37 (75.5%)
Female	12 (24.5%)
**Mean Height** (m)	
Male	1.69 ± 0.07
Female	1.62 ± 0.06
**Mean Weight** (kg)	
Male	60.5 ± 11.1
Female	50.1 ± 8.7
**Mean BMI** (kg/m^2^)	
Male	21 ± 3.6
Female	19.5 ± 2.6
**BMI** (n)	
<18 (Undernourished)	16 (32.6%)
≥18 to ≤22.9 (Normal)	18 (36.7%)
≥23 to ≤24.9 (Overweight)	10 (20.4%)
≥25 (Obese)	5 (10.2%)
**Disease Type**	
Ulcerative Colitis Crohn’s disease IBD-U	38 (77.6%) 6 (12.2%) 5 (10.2%)
Mean number of data entry days by patients using the app in 4 weeks	15.7 days

**Table 2:** Comparison between 24-h recall vs. IBD NutriCare app (Mean and standard deviation with 95% CI and Interclass correlation coefficient).

Variable	24 Hr Recall Mean ± SD (N = 49)	IBD NutriCare Mean ± SD (N = 49)	Difference in Mean ± SD	Intraclass Correlation Coefficient
Protein (g)	44.2 ± 17.9	44.7 ± 17.9	−0.57 ± 6.47	0.93 *
Fat (g)	32 ± 16.59	32 ± 16.80	0.37 ± 1.7	0.99 *
Carbohydrate (g)	204 ± 83.33	203 ± 86.62	1.59 ± 39.15	0.93 *
Energy (kcal)	1318 ± 447.53	1323 ± 463.62	−4.81 ± 210.78	0.94 *
Fiber (g)	29 ± 13.82	30 ± 12.94	0.61 ± 5.95	0.94 *
Cereals and millets (g)	188 ± 82.77	182 ± 85.59	6.42 ± 47.81	0.91 *
Grains and Legumes (g)	42.90 ± 34.58	43.65 ± 34.44	−0.78 ± 15.98	0.94 *
Vegetables (g)	190.27 ± 128.25	183.39 ± 92.48	6.88 ± 92.55	0.79 *
Fruits (g)	114.43 ± 129.55	128.70 ± 143.02	−14.27 ± 67.27	0.93 *
Milk (g)	257.59 ± 217.02	258.67 ± 233.73	−1.09 ± 105.58	0.94 *

* *p* < 0.001.

**Table 3:** Acceptability of the IBD NutriCare app.

Sl. No.	Statement (*n* = Number of Responders)	Agree/Strongly Agree *n* (%)	Undecided *n* (%)	Disagree/Strongly Disagree *n* (%)
1	Have you used any previous apps before? (*n* = 49)	44 (90)	0	5 (10)
2	Was the learning process regarding app usage easy? (*n* = 49)	45 (92)	3 (6)	1(2)
3	Did you find the app convenient? (*n* = 49)	47 (96)	2 (4)	0
4	Were the food items easily identifiable in the app? (*n* = 49)	40 (82)	4 (8)	5(10)
5	Was the app accurate? (*n* = 49)	42 (86)	6 (12)	1(2)
6	Easy to estimate portion size (*n* = 49)	42 (86)	3 (6)	4 (8)
**Sl. No**	**Comparing two methods (*n* = 49)**	**App**	**24-h recall**	**Undecided**
1	Preferred Method	44 (90)	5(10)	0
2	More convenient method	42 (86)	5 (10)	2 (4)
3	Found Easier to complete	43 (88)	4 (8)	2 (4)
4	More accurate	40 (82)	5 (10)	4 (8)
5	Less time consuming	46 (94)	3 (6)	0
6	Easy to remember	44(90)	4 (8)	1 (2)
7	Disease awareness	42 (86)	5 (10)	2 (4)

## Data Availability

The data will be made available on reasonable request.

## References

[1] Kedia S., Ahuja V. (2018). Is the emergence of inflammatory bowel disease a prime example of “the third epidemiological transition”?. Indian J. Gastroenterol..

[2] Singh P., Ananthakrishnan A., Ahuja V. (2017). Pivot to Asia: Inflammatory bowel disease burden. Intest. Res..

[3] Mak W.Y., Zhao M., Ng S.C., Burisch J. (2020). The epidemiology of inflammatory bowel disease: East meets west. J. Gastroenterol. Hepatol..

[4] Kaplan G.G., Windsor J.W. (2021). The four epidemiological stages in the global evolution of inflammatory bowel disease. Nat. Rev. Gastroenterol. Hepatol..

[5] Park J., Cheon J.H. (2021). Incidence and Prevalence of Inflammatory Bowel Disease across Asia. Yonsei Med. J..

[6] Kedia S., Ahuja V. (2017). Epidemiology of Inflammatory Bowel Disease in India: The Great Shift East. Inflamm. Intest. Dis..

[7] Levine A., Boneh R.S., Wine E. (2018). Evolving role of diet in the pathogenesis and treatment of inflammatory bowel diseases. Gut.

[8] Ananthakrishnan A.N. (2020). Impact of Diet on Risk of IBD. Crohn’s Colitis 360.

[9] Serrano-Moreno C., Brox-Torrecilla N., Arhip L., Romero I., Morales Á., Carrascal M.L., Cuerda C., Motilla M., Camblor M., Velasco C. (2022). Diets for inflammatory bowel disease: What do we know so far?. Eur. J. Clin. Nutr..

[10] Fitzpatrick J.A., Melton S.L., Yao C.K., Gibson P.R., Halmos E.P. (2022). Dietary management of adults with IBD—The emerging role of dietary therapy. Nat. Rev. Gastroenterol. Hepatol..

[11] Roncoroni L., Gori R., Elli L., Tontini G.E., Doneda L., Norsa L., Cuomo M., Lombardo V., Scricciolo A., Caprioli F. (2022). Nutrition in Patients with Inflammatory Bowel Diseases: A Narrative Review. Nutrients.

[12] Levine A., Rhodes J.M., Lindsay J.O., Abreu M.T., Kamm M.A., Gibson P.R., Gasche C., Silverberg M.S., Mahadevan U., Boneh R.S. (2020). Dietary Guidance From the International Organization for the Study of In-flammatory Bowel Diseases. Clin. Gastroenterol. Hepatol..

[13] Shafiee N.H., Manaf Z.A., Mokhtar N.M., Ali R.A. (2021). Anti-inflammatory diet and inflammatory bowel disease: What clinicians and patients should know?. Intest. Res..

[14] Carter M.C., Burley V.J., Nykjaer C., Cade J.E. (2013). ‘My Meal Mate’ (MMM): Validation of the diet measures captured on a smartphone application to facilitate weight loss. Br. J. Nutr..

[15] India to Have 1 bn Smartphone Users by 2026: Deloitte Study. https://www.businesstoday.in/technology/story/india-to-have-1-bn-smartphone-users-by-2026-deloitte-study-323519-2022-02-22.

[16] Elamin S., Cohen J. (2021). Telenutrition for Inflammatory Bowel Disease: A Tipping Point for Dietary Wellness. Crohn’s Colitis 360.

[17] de Jong M., van der Meulen-de Jong A., Romberg-Camps M., Degens J., Becx M., Markus T., Tomlow H., Cilissen M., Ipenburg N., Verwey M. (2017). Development and feasibility study of a telemedicine tool for all patients with IBD: MyIBD-coach. Inflamm. Bowel Dis..

[18] Bosworth T., Kling J. (2019). AGA Tech Summit Telemedicine Proving to Be an Efficient Platform for Delivering Nutritional Services.

[19] Walmsley R.S., Ayres R.C.S., Pounder R.E., Allan R.N. (1998). A simple clinical colitis activity index. Gut.

[20] Harvey R.F., Bradshaw J.M. (1980). A simple index of crohn’s-disease activity. Lancet.

[21] Klein S. (2002). A primer of nutritional support for gastroenterologists. Gastroenterology.

[22] Cross R.K., Cheevers N., Finkelstein J. (2009). Home telemanagement for patients with ulcerative colitis (UC HAT). Dig. Dis. Sci..

[23] Yin A.L., Hachuel D., Pollak J.P., Scherl E.J., Estrin D. (2019). Digital Health Apps in the Clinical Care of Inflammatory Bowel Disease: Scoping Review. J. Med. Internet Res..

[24] Ambrosini G.L., Hurworth M., Giglia R., Trapp G., Strauss P. (2018). Feasibility of a commercial smartphone application for dietary assessment in epidemiological research and comparison with 24-h dietary recalls. Nutr. J..

[25] Sood A., Ahuja V., Kedia S., Midha V., Mahajan R., Mehta V., Sudhakar R., Singh A., Kumar A., Puri A.S. (2019). Diet and inflammatory bowel disease: The Asian Working Group guidelines. Indian J. Gastroenterol..

[26] Shoda R., Matsueda K., Yamato S., Umeda N. (1996). Epidemiologic analysis of Crohn disease in Japan: Increased dietary intake of n-6 polyunsaturated fatty acids and animal protein relates to the increased incidence of Crohn disease in Japan. Am. J. Clin. Nutr..

[27] Wellard-Cole L., Chen J., Davies A., Wong A., Huynh S., Rangan A., Allman-Farinelli M. (2019). Relative Validity of the Eat and Track (EaT) Smartphone App for Collection of Dietary Intake Data in 18-to-30-Year Olds. Nutrients.

[28] Rollo M.E., Ash S., Lyons-Wall P., Russell A.W. (2015). Evaluation of a Mobile Phone Image-Based Dietary Assessment Method in Adults with Type 2 Diabetes. Nutrients.

[29] Sharp D.B., Allman-Farinelli M. (2014). Feasibility and validity of mobile phones to assess dietary intake. Nutrition.

[30] Sahu P., Kedia S., Ahuja V., Tandon R.K. (2021). Diet and nutrition in the management of inflammatory bowel disease. Indian J. Gastroenterol..

[31] https://economictimes.indiatimes.com/nri/migrate/at-18-million-india-has-the-worlds-largest-diaspora-population/articleshow/80290768.cms.

